# Supplementary material for: Different Time Courses of Mono- and Bi-Liganded Bursts of Channel Openings of Adult nAChR Molecules Formed by the Reactions of Transmembrane Regions
Source: Cells. 2024 Dec 17;13(24):2079. doi: 10.3390/cells13242079 (PMC11674366; doi:10.3390/cells13242079)
Supplement: Supplementary file 1 [file cells-13-02079-s001.zip › cells-3337616-supplementary.pdf]

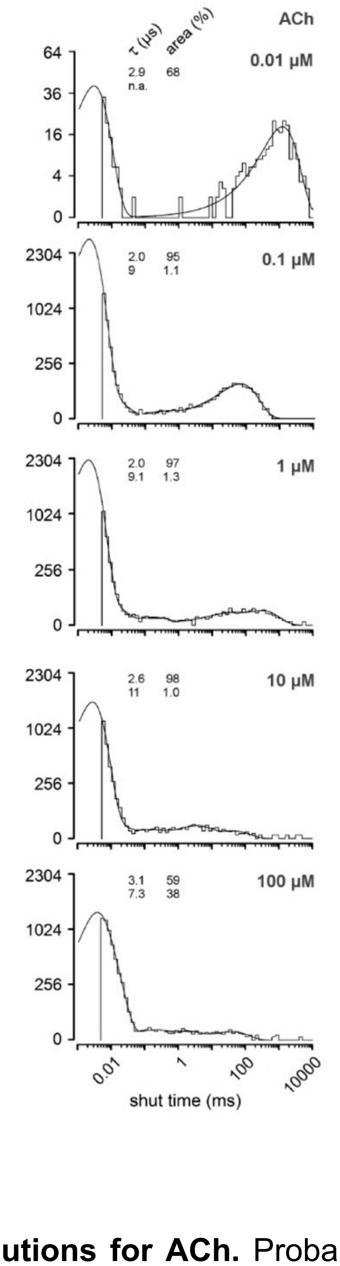

Figure S1. Shut time distributions for ACh. Probability density functions fitted to shut time distributions from recordings with 0.01 to 100 µM ACh. ACh concentration increases from top to bottom, as indicated on the right. Ordinates give the number of events per bin and the axis of abscissae event durations on a log scale. The parameters τ and areas of each fit are given in the upper left of each plot. Each shut-time distribution contains a prominent first peak at about 2 µs. For clarity, only the two shortest shut time components are shown.

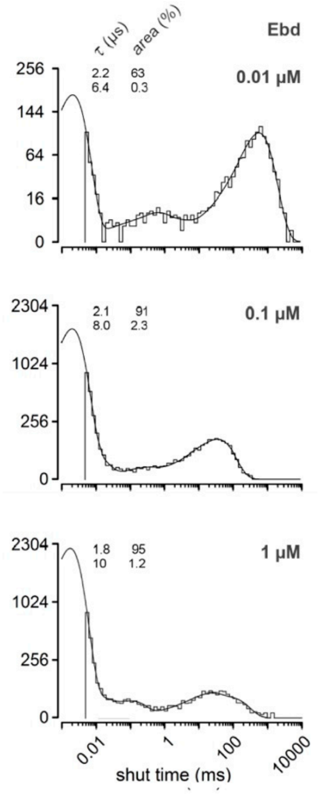

Figure S2. Shut time distributions for Ebd. Probability density functions fitted to shut time distributions from recordings at 0.01 to 1 μM epibatidine (Ebd). It is the same arrangement as in Figure 3. Shut-time distributions again contain a prominent first peak at about 2 μs, similar to ACh recordings. For clarity, only the two shortest shut time components are shown.

Table S1. Mean burst durations with long t_{crit} for ACh and Ebd. Burst durations of adult mouse muscle type nAChRs with ACh or Ebd and tcrit = 100 μs. Mean values of time constants with standard deviations from fits of distributions from 3 recordings are given in milliseconds. Mean component areas are shown in %. For clarity, single openings were removed from burst length distributions.

	short (αε) bursts		long (αδ+αε) bursts	
ACh (μM)	τ_{b1} (ms)	area (%)	τ_{b2} (ms)	area (%)
0.01	0.46±0.06	100±0		
0.1	0.53±0.16	36±20	12.9±5.1	64±20
1			14.7±1.3	100±0
10			14.7±7.7	100±0
100			18.3±9.0	100±0
Ebd (μM)				
0.01	0.28±0.04	71±16	10.1±1.0	30±16
0.1	0.26±0.03	46±14	7.5±2.5	54±14
1	0.15±0.06	9±6	6.1±1.3	91±6

Table S2. Mean open periods of partially blocked receptors. Open periods of adult mouse muscle type nAChRs at the indicated ACh and Ebd concentrations after blockade by CTx (n=3 measurements per concentration) or Wtx (n=4 measurements per concentration). Mean values of time constants with standard deviations from fits of distributions are given in microseconds. Mean component areas are shown in %. n.a., the component occurred in one measurement only. Thus, no mean value can be given.

	very short openings (τ_{o1})		short openings (τ_{o2})		intermediate openings (τ_{o3})	
ACh (μM) **+ CTx**	τ_{o1} (μs)	area (%)	τ_{o2} (μs)	area (%)	τ_{o3} (μs)	area (%)
0.1 μM			35±9	100±0		
1μM			37±9	100±0		
10μM			37±3	100±0		
100 μM	3.7±1.6	31±16	29±0	67±17	135±28	2±2
ACh (μM) **+ WTx**						
10μM	n.a.	n.a	15±7	84±33		
Ebd (μM) **+ CTx**						
0.1 μM	n.a.	n.a	36±0	93±12		
